# Pulmonary and Cardiac Function in Asymptomatic Obese Subjects and Changes following a Structured Weight Reduction Program: A Prospective Observational Study

**DOI:** 10.1371/journal.pone.0107480

**Published:** 2014-09-18

**Authors:** Matthias Held, Maria Mittnacht, Martin Kolb, Sabine Karl, Berthold Jany

**Affiliations:** 1 Medical Mission Hospital, Academic Teaching Hospital, Julius Maximilian University of Würzburg, Department of Internal Medicine, Würzburg, Germany; 2 Firestone Institute for Respiratory Health, Department of Medicine, Pathology & Molecular Medicine, McMaster University, Hamilton, ON, Canada; 3 Institute of Mathematics, Julius Maximilian University of Würzburg, Würzburg, Germany; Dasman Diabetes Institute, Kuwait

## Abstract

**Background:**

The prevalence of obesity is rising. Obesity can lead to cardiovascular and ventilatory complications through multiple mechanisms. Cardiac and pulmonary function in asymptomatic subjects and the effect of structured dietary programs on cardiac and pulmonary function is unclear.

**Objective:**

To determine lung and cardiac function in asymptomatic obese adults and to evaluate whether weight loss positively affects functional parameters.

**Methods:**

We prospectively evaluated bodyplethysmographic and echocardiographic data in asymptomatic subjects undergoing a structured one-year weight reduction program.

**Results:**

74 subjects (32 male, 42 female; mean age 42±12 years) with an average BMI 42.5±7.9, body weight 123.7±24.9 kg were enrolled. Body weight correlated negatively with vital capacity (R = −0.42, *p<0.001*), FEV1 (R = −0.497, *p<0.001*) and positively with P 0.1 (R = 0.32, p = 0.02) and myocardial mass (R = 0.419, *p* = 0.002). After 4 months the study subjects had significantly reduced their body weight (−26.0±11.8 kg) and BMI (−8.9±3.8) associated with a significant improvement of lung function (absolute changes: vital capacity +5.5±7.5% pred., *p<0.001*; FEV1+9.8±8.3% pred., *p<0.001*, ITGV+16.4±16.0% pred., *p<0.001*, SR tot −17.4±41.5% pred., *p<0.01*). Moreover, P0.1/Pimax decreased to 47.7% (p<0.01) indicating a decreased respiratory load. The change of FEV1 correlated significantly with the change of body weight (R = −0.31, *p* = 0.03). Echocardiography demonstrated reduced myocardial wall thickness (−0.08±0.2 cm, p = 0.02) and improved left ventricular myocardial performance index (−0.16±0.35, p = 0.02). Mitral annular plane systolic excursion (+0.14, p = 0.03) and pulmonary outflow acceleration time (AT +26.65±41.3 ms, p = 0.001) increased.

**Conclusion:**

Even in asymptomatic individuals obesity is associated with abnormalities in pulmonary and cardiac function and increased myocardial mass. All the abnormalities can be reversed by a weight reduction program.

## Introduction

The worldwide prevalence of obesity is rising [1]. Obesity leads to increasing morbidity [1], [2], [3] and mortality [4], a loss of potential and quality adjusted years of life and is a major economic challenge for health care systems [5]. It contributes to an increasing risk for a large variety of diseases including arterial hypertension, diabetes mellitus, dyslipidemia, coronary artery disease, stroke, cancer and obstructive sleep apnea [1]. A negative influence of obesity on diastolic and systolic ventricular function was reported as well as “obesity cardiomyopathy” [6], [7]. Obesity is also an independent predictor for the development of pulmonary hypertension in patients with diastolic left ventricular dysfunction [8]. In patients with obstructive sleep apnea, body weight correlates with pulmonary artery pressure [9]. Further, obesity can cause obesity hypoventilation syndrome (OHS) [10], [11] and may contribute to pulmonary hypertension in these patients [10], [11]. In subjects with OHS and severe PH, pulmonary artery pressure is correlated to body mass index, pCO_2_ and reduced power of breathing [11]. However, not all obese subjects develop obesity hypoventilation syndrome [12], and neither all patients with OHS are affected by pulmonary hypertension [13]. The cardiac and pulmonary function in asymptomatic obese subjects is not well studied.

Obesity can affect pulmonary function in multiple ways. Static lung volumes and maximal power of breathing is reduced. Airway resistance and work of breathing is increased [14], [15]. A contributory role of these abnormalities to the pathophysiology of asthma and pulmonary arterial hypertension in obese patients is likely [16]–[24]. Humoral interactions, e.g. leptin resistance contributing to hypoventilation [29], and a major role of adiponectine in the pathophysiology of PAH and asthma have been discussed [25]–[28]. However, it remains unclear whether body fat contributes to functional abnormalities in the cardiopulmonary system via an underlying systemic inflammatory process or if ventilation and pulmonary circulation are only influenced by the mechanical influences of increased body fat [30]. Because interactions and the sequence of events remain unclear, it is of great interest to determine possible early events on the path to clinically evident disease. It is unclear whether obesity has an impact on lung and cardiac function in asymptomatic subjects.

Interventional programs are promising tools to reduce obesity associated morbidity and mortality. The benefit of surgically induced weight loss on lung and cardiac function has been convincingly demonstrated [13], [31]. In contrast, there are few studies that have assessed the effect of structured weight reduction programs on pulmonary function and echocardiographically measured cardiac function, blood pressure and pulmonary hemodynamics assessed by echocardiography or right heart catheterization and they have shown conflicting results [32]–[34]. Especially the effect of dietary induced weight loss on lung and cardiac function of asymptomatic subjects has not been studied.

We studied and report the effect of a weight reduction program which is based on the use of a formula diet on lung and cardiac function. It includes a supervision of all participants by a multidisciplinary team of physicians, dietary specialists, physiotherapists and psychologists specialized in the long-term treatment of obese patients.

### Objective

We aimed to determine the influence of body weight on lung and cardiac function in asymptomatic obese adults and to evaluate the effect of a structured weight reduction program on lung and cardiac function.

We show that even in asymptomatic individuals obesity is associated with cardiopulmonary functional abnormalities and these abnormalities can be reversed by weight reduction.

## Methods

In a prospective observational study lung function tests and echocardiography were performed in 74 subjects who underwent an interdisciplinary 52-week weight reduction program. The structure of this weight loss program addressing people with a BMI>30 kg/m^2^ was described before [35]. It consists of a one-week run-in-period, a twelve week fasting period followed by an eight week changeover and a 31-week stabilization period. The study was approved by our local Ethics Committee. Baseline data were collected during the one-week run-in-period. A follow-up examination 16 weeks after start of the dietary program was offered to all patients. The time point four weeks after completion of the fasting period was chosen to analyze the effect of potential maximum weight loss on cardiopulmonary function.

### Study subjects

Study subjects were recruited from 83 consecutive subjects who registered for the structured dietary program at Medical Mission Hospital between January 2009 and May 2012. 74 patients gave written informed consent and were studied at baseline and at follow-up 12 weeks later. Nine patients declined to participate. Eight patients who had stopped the dietary program during the first twelve weeks were not examined at the 16-week follow-up. Some patients did not complete all measurements of the follow-up examination. Only patients with data available from both baseline and three month follow-up period were included for analysis of changes in each parameter.

### Procedures

Bodyplethysmography including measurement of transfer factor (Masterscreen Body/Diff CareFusion, Germany) was performed according to the European Respiratory Society Statement [36]. Inspiratory mouth pressures were measured as described [37]–[41]. Echocardiography (Vivid7, GE Medical Systems, Solingen, Germany) was performed to measure left and right ventricular function, myocardial mass, systolic right ventricular pressure, right ventricular outflow tract acceleration time and to rule out cardiac shunts and significant valve pathology as recommended [42], [43]. We further analyzed the available data of Bioelectrical Impedance analysis, which was introduced into the weight reduction program during the study. Bioelectrical impedance analysis (BIA 2000-S, Data Input GmbH Darmstadt, Germany) was performed as recommended and according to the manufacturers guideline [44], [45]. The same diagnostic tests used at baseline were performed at the follow-up.

### Statistical analysis

Statistical analysis was done using the program SPSS, Version 19.0 (IBM SPSS Statistics). Data are presented as mean and standard deviation. Baseline and follow-up values were compared and significance was evaluated by the paired t-test and assumed if *p*-value was <0.05. Pearson correlation of the parameters was calculated. Statistical significance was assumed if p-value was <0.05.

## Results

### Anthropometric data and bioelectrical impedance analysis

**Figure 1:**
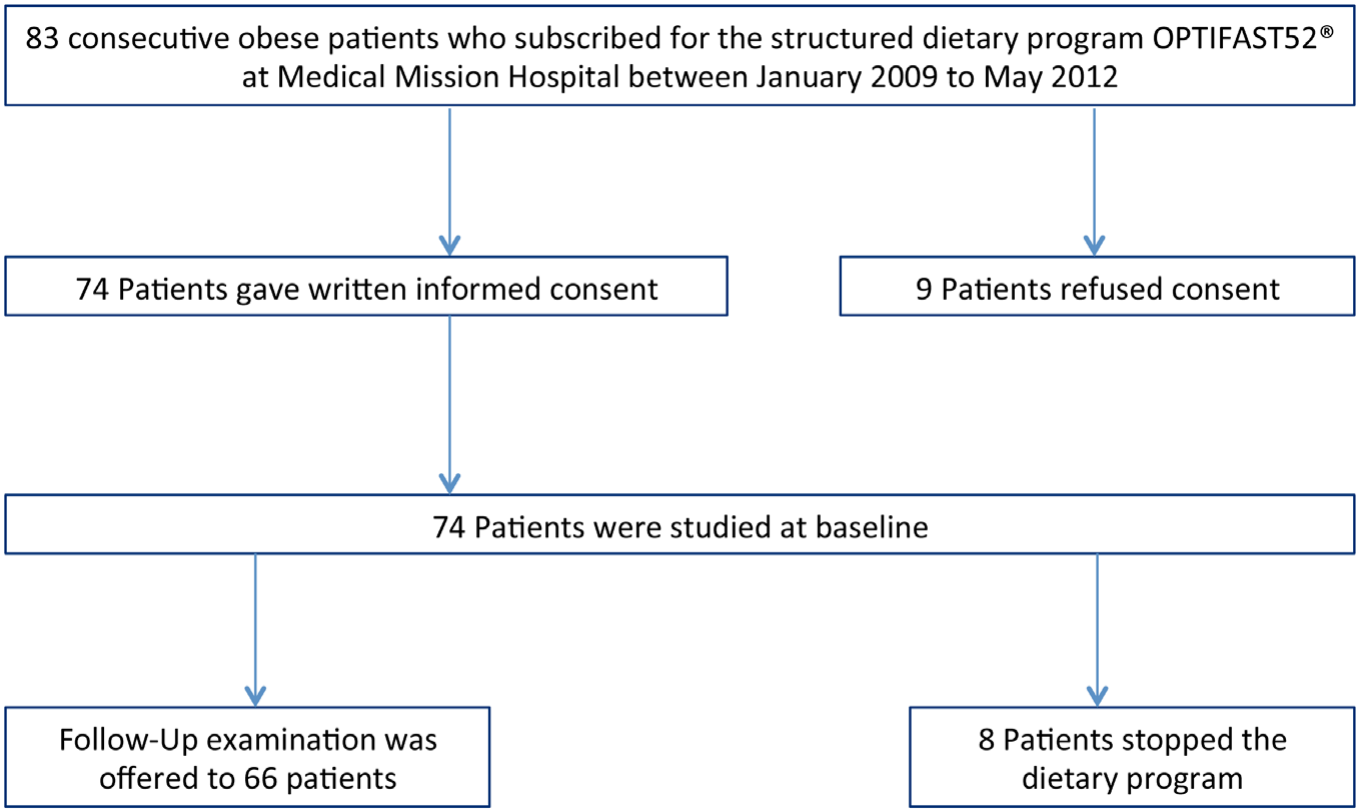
Subject selection for the analysis.

The subjects were selected as shown in figure 1. Table 1 summarizes the anthropometric data of the 74 individuals at baseline. The results of the bioelectric impedance analysis at baseline are shown in Table S1. The cohort comprised 32 males and 42 females. Mean age was 43±12 years. The subjects presented with severe obesity with a mean body-mass-index (BMI) of 42.5±7.9 kg/m^2^ and a mean body weight of 123.7±24.9 kg. The waist-to-hip ratio was 0.94±0.1. Body fat was 55.8±16.6 kg and 44.4±8.4% respectively. Resting metabolic rate was 1756.4±293.9 kcal. Body fluid was calculated with 50.7±11.5 l. The majority of subjects presented without dyspnea: NYHA I = 68/74, NYHA II = 6/74, NYHA III = 0/74, NYHA IV = 0/74. Seven of the 74 subjects were smokers. Comorbidities are shown in table S2.

**Table 1:** Anthropometric data.

Parameter		N = 74, Value Mean ± SD
**Anthropometric data**		
female/male	74	32/42
Age (years)	74	43±12
BMI (kg/m^2^)	74	42.5±7.9
Body weight (kg)	74	123.7±24.9
Waist/hip ratio	74	0.94±0.1
Systolic blood pressure	74	133±14
Diastolic blood pressure	74	89±9
Heart rate	74	73±9
NYHA Class I/II/III/IV	74	68/6/0/0

Data are shown as mean ± standard deviation.

### Baseline data

**Figure 2:**
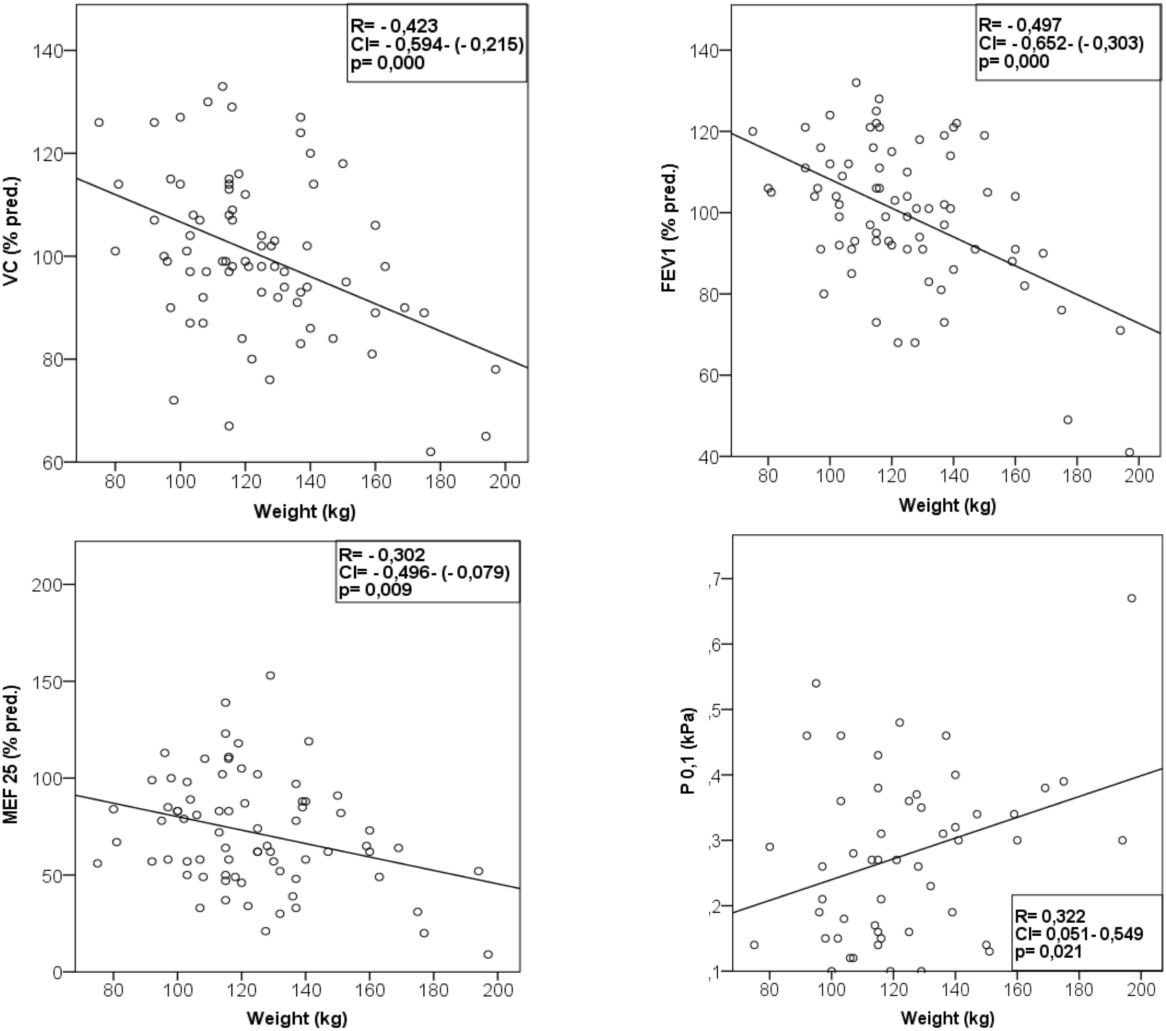
Correlations of body weight with pulmonary function. VC = vital capacity. FEV1 = forced expired volume at one second. MEF 25 = Mean expiratory flow 25. P 0.1 = mouth occlusion pressure at 0.1 second.

The results of the pulmonary function tests are summarized in table 2. Mean expiratory flow 25, intrathroracic gas volume (ITGV), total lung capacity and transfer factor of the lung measured by the single breath method (TLCO-SB) were mildly decreased compared to the, not weight specific normal values, published by Quanjer for a cohort of subjects aged 18–70 years with a height of 1.45–1.95 m [36]. P0.1 and P0.1/Pimax were increased compared to a cohort with normal weight reported by Koch et al [38] and non weight-specific reference values proposed by the German Airway League [39] based on data published by Hautmann and Windisch [40], [41] indicating an increased respiratory load. There was a significant negative correlation between body weight and vital capacity (R = −0.42, *p*<0.001), FEV_1_ (R = −0.49, *p*<0.001), MEF_25_ (R−0.30, *p* = 0.009) (Fig. 2) and FEV_1_/VC (R = 0.37, *p* = 0.001). Body weight (R = 0.32, p = 0.02) (Figure 2) and body fat (R = 0.316, p = 0.03) was positively correlated to P0.1. FEV_1_ and MEF_25_ were negatively correlated to body fat. We found a significant negative correlation of waist-to-hip-ratio with vital capacity (R = −0.43, p<0.001) and with total lung capacity (R = −0.34, p = 0.003).

**Table 2:** Baseline data: Bodyplethysmography including mouth occlusion pressure and echocardiography.

	N	Baseline (Mean ± SD)
Bodyplethysmography		
VC (% pred.)	74	100.4±15.6
FEV_1_ (% pred.)	74	99.8±17.7
FEV_1_/VC (%)	74	79.2±7.7
MEF 25 (% pred.)	74	71.9±28.6
ITGV (% pred.)	74	91.2±16.5
RV (% pred.)	74	102.1±32.7
TLC (% pred.)	74	102.0±12.2
TLC-He (% pred)	30	90.0±11.0
SR tot (% pred.)	74	109.1±63.5
TLCO SB (% pred.)	47	87.3±14.0
P0.1 (kPa)	51	0.28±0.13
PI max (kPa)	36	6.8±2.9
P0.1/PI max	29	0.05±0.05
BF (1/min)	35	18.0±5.5
Echocardiography		
IVSd (mm)	54	10.9±2.5
LVPWd (mm)	54	10.6±2.3
EF biplan Simpson (%)	53	63.1±7.2
LIMP	46	0.54±0.28
MAPSE (cm)	51	1.8±0.4
E/E’	54	6.74±1.67
TAPSE (mm)	54	28.8±5.1
RIMP	51	0.40±0.22
RA area (cm^2^)	54	13.9±5.5
RV (cm)	54	3.2 0.7
e/e’	51	4.82±1.67
TDI-TVA (cm/s)	51	14.5±3.0
RVSP (mmHg)	7	20.4±8.6
PV AccT (ms)	50	117.8±29.6

Data are shown as mean ± standard deviation.

Echocardiographic data at baseline are shown in table 2. We found an increased left and right myocardial performance index compared to the normal values proposed by the American (ASE) and European Society of Echocardiography (ESE) [43] that are not weight specific. e/e’ was increased compared to these normal values indicating diastolic dysfunction of the right ventricle. Mean diameter of the interventricular septum and posterior wall of the left ventricle were mildly enlarged in comparison to the normal values recommended by ASE and ESE published by Lang et al [42]. However, diameter of all cardiac chambers, ejection fraction, mitral flow pattern and tissue Doppler parameter of the left ventricle were normal.

**Figure 3:**
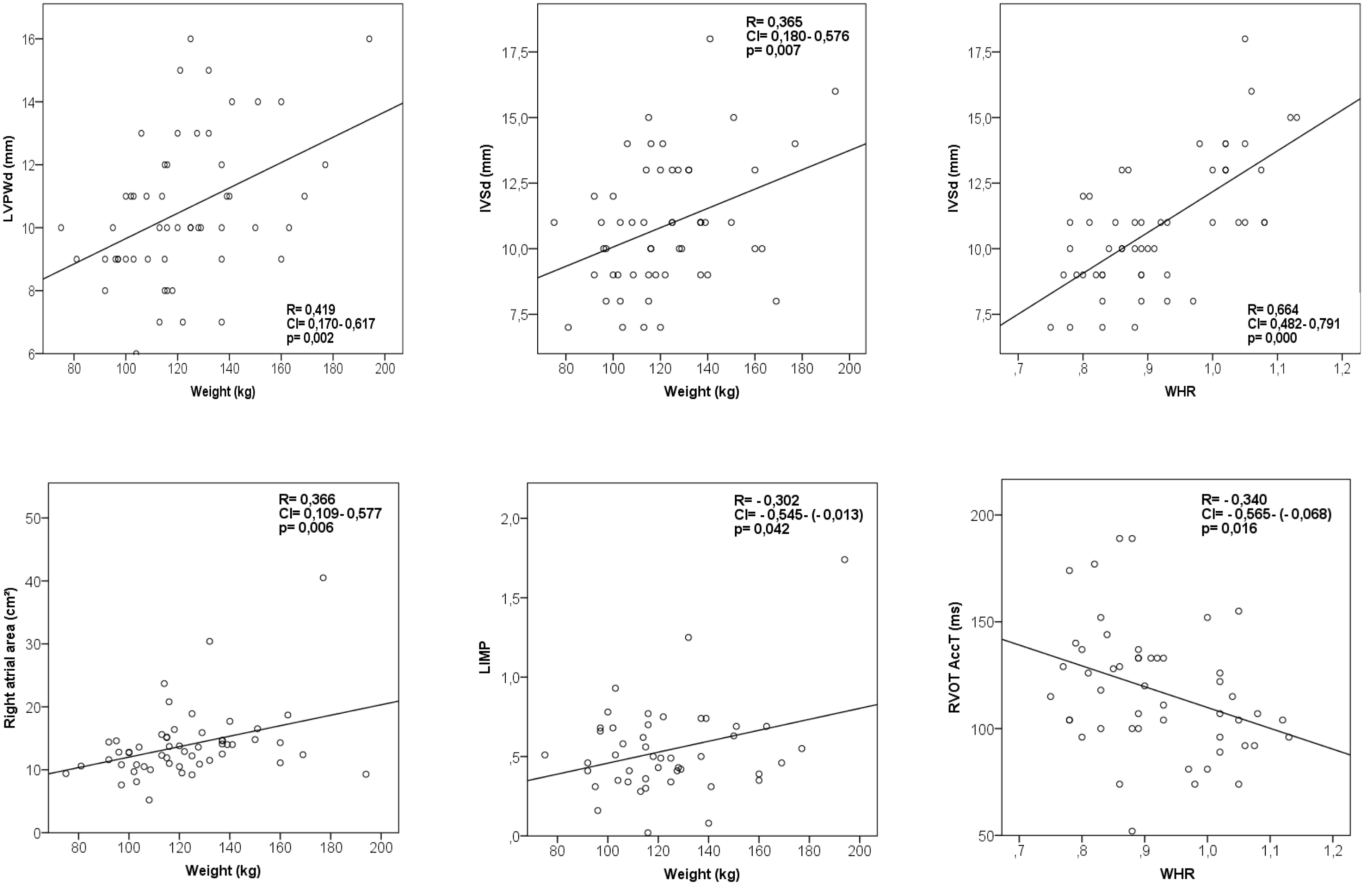
Correlations of body weight with cardiac function and myocardial wall thickness. LVPWd = diastolic left ventricular posterior wall diameter; IVSd = diastolic interventricular septal wall thickness; LIMP = left myocardial performance index; RVOT AccT = acceleration time of flow in the right ventricular outflow tract.

We found a positive correlation between body weight and myocardial wall thickness (IVSd, R = 0.37, p = 0.007; LPWd (R = 0.42, *p = 0.002*), body weight and left myocardial performance index (R = 0.30, *p* = 0.04) and body weight and right atrial area (R = 0.37, p = 0.006), (Figure 3). Waist-to-hip ratio was positively correlated with myocardial wall thickness (IVSd, R = 0.66, p<0.001) and acceleration time in the right ventricular outflow tract (R = −0.34, p = 0.02; Figure 3).

### Follow-Up data

Follow-up data and absolute changes from baseline are shown in table 3. After 4 months of the dietary program, body weight decreased dramatically (−26.0±11.8 kg, *p*<0.001), as well as BMI (−8.9±3.8, *p*<0.001) and body fat (−18.5±7.7, p<0.001). Table 3 shows the significant increase of lung volumes measured by bodyplethysmography and spirometric flow parameters. P0.1/Pimax decreased indicating improving breathing capacity.

**Table 3:** Baseline and follow-up data, absolute changes and p values.

	N	Baseline(Mean ± SD)	Follow-Up(Mean ± SD)	abs. change(Mean ± SD)	p-value
Weight, Height and Waist to hip ratio					
Weight (kg)	55	125.6±24.5	99.6±17.9	−26.0±11.8	<0,001
BMI (kg/m^2^)	55	43.4±8.1	34.5±6.3	−8.9±3.8	<0,001
WHR	62	0.93±0.11	0.90±0.09	−0.03±0.1	<0,001
Bodyplethysmography					
VC (% pred.)	51	98.5±15.7	104±13.7	+5.5±7,5	<0,001
FEV_1_ (% pred.)	51	97.7±18.8	107.5±17.4	+9.8±8.3	<0,001
FEV_1_/VC (%)	50	78,9±8.4	80.9±7.9	+2.0±4.9	0,006
MEF 25 (% pred.)	51	68.7±29,008	82.3±30.5	+13.6±20.6	<0,001
ITGV (% pred.)	51	90.6±17.2	107.0±21.0	+16.4±16.0	<0,001
RV (% pred.)	50	100.6±32.9	87.2±38.0	−13.4±35.4	0,01
TLC (% pred.)	51	100.78±12.1	101.1±12.1	+0,25±11.0	0,86
SR tot (% pred.)	51	110.7±63.0	93.3±36.6	−17.4±41.5	<0,01
TLCO SB (% pred.)	32	89.7±13.1	91.84±13.2	+2,2±10.5	0,26
TLCO/VA (% pred.)	32	106.2±13.8	101.9±13.1	−4.3±9.7	0,018
TLC-He (% pred.)	30	89.9±10.6	95.2±10.0	+5.3±6.0	<0,001
P0.1 (kPa)	35	0.31±0.12	0.23±0.11	−0.07±1.0	<0,001
PI max (kPa)	36	6.9±3.0	7.2±2.4	+0.3±2,3	0.59
P0.1/PI max	29	0.06±0.05	0.04±0.03	−0.02±0.05	<0.01
BF (1/min)	35	19.1±5.8	18.2±4.6	−0.9±5.5	0.35
Echocardiography					
IVSd (cm)	35	1.05±0.23	0.97±0.18	−0.08±0.20	0.02
LVPWd (cm)	35	1.05±0.24	0.97±0.19	−0.08±0.20	0.03
EF biplan Simpson (%)	35	64±6.8	62.9±7.2	−1.06±9.2	0.5
LIMP	27	0.61±0.32	0.45±0.31	−0.16±0.35	0.02
MAPSE (cm)	34	1.81±0.35	1.95±0.40	+0.14±0.36	0.03
LVEI	34	0.96±0,09	0,93±0.10	−0.03±0.10	0,09
e/e’	35	5.07±1.81	4.76±1.19	−0.31±1.48	0,22
TDI-TVA (cm/s)	35	14.3±2.9	13.1±2.62	−1.2±3.1	0,03
RVSP (mmHg)	2	28.3±11.7	20.5±4.5	−7.8±6.7	0,35
PV AccT (ms)	31	123.0±29.4	149.6±37.9	+26.6±41.3	0,001
BIA measurements					
Resting metabolic rate (Kcal)	49	1750±295	1554±171	−195±197	<0,001
Phase angle (°)	49	5.99±0.75	5.36±0.86	−0.63±0.99	<0,001
Body fluid (l)	49	50.6±11.4	45.1±8.5	−5,5±4,2	<0,001
Lean body mass (kg)	49	69.1±15.6	61.7±11.6	−7.4±5.8	<0,001
ECM (kg)	49	33.2±7.1	32.0±7.3	−1.2±2.5	0,002
BCM (kg)	49	35.9±9.3	29.7±5.4	−6.2±6.2	<0,001
ECM/BCM-Index	49	0.94±0.13	1.08±0.18	+0.14±0.19	<0,001
Cell amount (%)	49	51.7±3.6	48.4±4.3	−3.3±4.7	<0,001
Body fat (kg)	49	57.4±16.6	38.9±15.3	−18.5±7.7	<0,001
Body fat (%)	49	45.1±8.0	37.8±9.6	−7.3±4.9	<0,001
Body fat corr. (kg)	49	59.1±16.6	37.8±14.6	−21.3±7	<0,001

Data are shown as mean ± standard deviation. This table shows data of subjects with corresponding data at both timepoints, baseline and follow-up examination.

**Figure 4:**
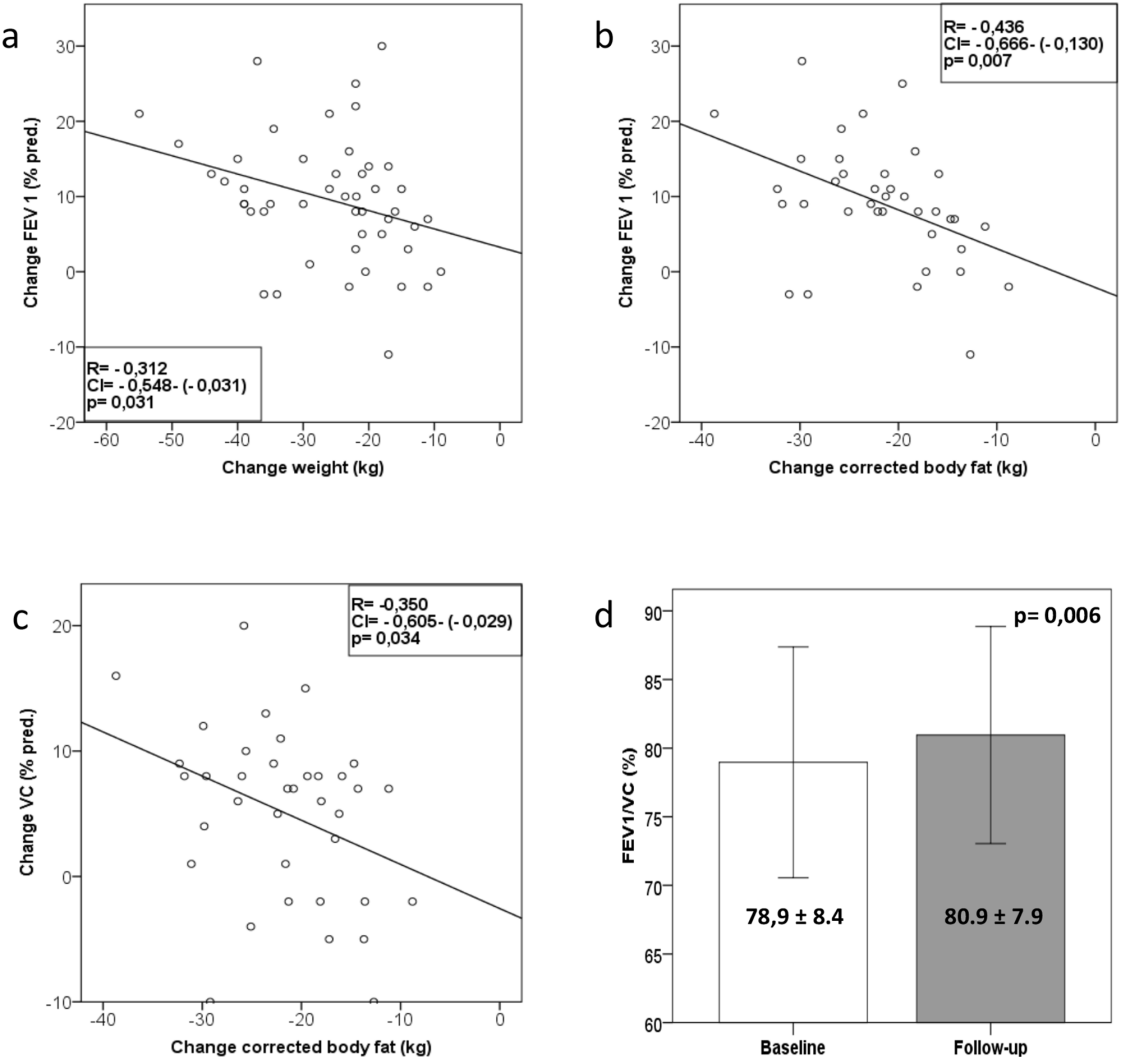
Correlations of changes of pulmonary function with changes of body weight and changes of body fat and change of FEV1/VC from baseline to follow-up. a–c: Correlations of changes of pulmonary function with changes of body weight and changes of body fat (a–c) d: Change of FEV1/VC (%) ratio from baseline to follow-up (d), (data as mean ± SD), p = 0.006. VC = vital capacity. FEV1 = forced expired volume at one second.

The change of vital capacity (R = −0.35, *p* 0.03) and FEV1 (R = −0.44, <*p* 0.01) negatively correlated with the change of body fat. (Figure 4). The change of FEV_1_ (R = –0.31, p = 0.03) (Figure 4), but not of vital capacity was correlated to the change of body weight.

Additionally we noticed a decrease of myocardial wall thickness (−0.08±0.20 cm, *p* = 0.02), an improvement of left ventricular myocardial performance index (−0.16±0.35, *p* = 0.02), and mitral annular plane systolic excursion MAPSE (+0.14 cm±0.36, *p = 0.03*). Pulmonary outflow acceleration time increased (AT +26.6 ms±41.3, *p = 0.001*) indicating a decrease of pulmonary vascular resistance.

**Figure 5:**
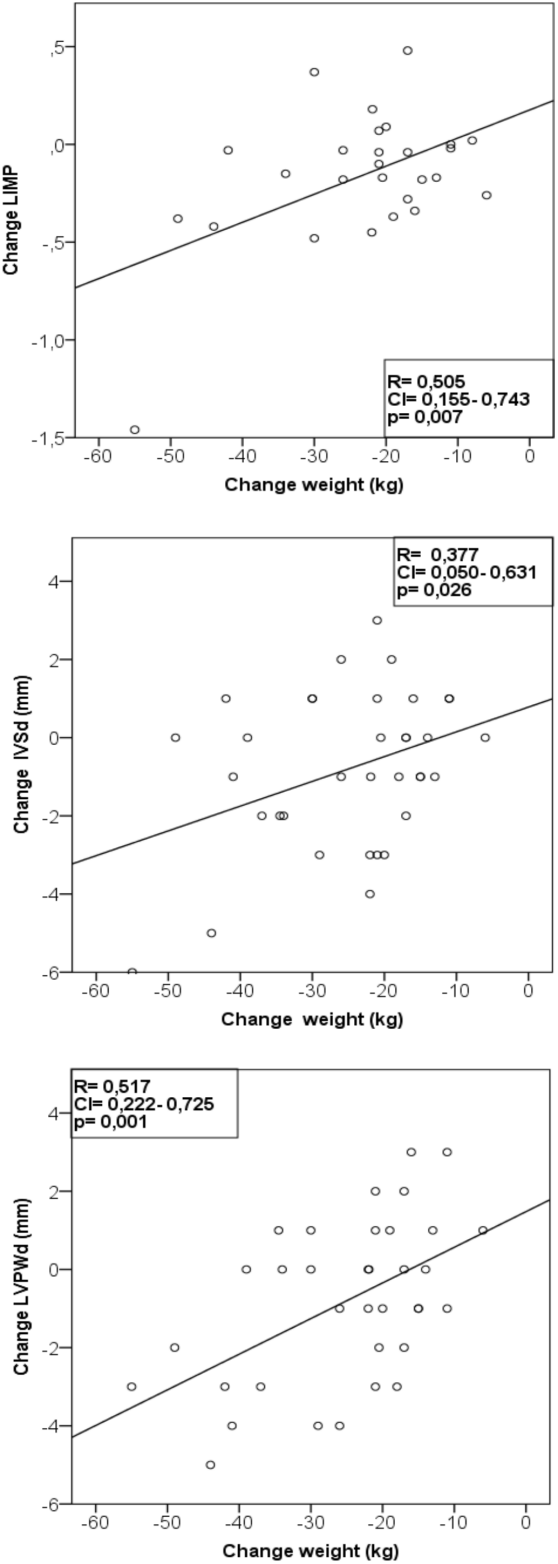
Correlations of changes of left myocardial performance index and myocardial wall thickness with changes of body weight. LIMP = left myocardial performance index. LVPWd = diastolic left ventricular posterior wall diameter; IVSd = diastolic interventricular septal wall thickness.

The decrease of myocardial wall thickness and the improvement of left myocardial performance index were positively correlated with the decrease of body weight (Figure 5).

Furthermore, the subjects with a history of asthma (n = 5) showed an absolute increase of FEV_1_ (+13.8%, p<0.001). An absolute decrease of residual volume (RV) of −15.8% led to an absolute increase of VC (+10.6%, p = 0.005). Due to the increase of FEV_1_ and VC the absolute increase of FEV_1_/FV (+1.1%, p = 0.6) was not significant (Table S3).

The patients with OSA showed an absolute increase of FEV_1_ (+12.3%, p = 0.02) and VC (+10.3%, p = 0.007). P 0.1 decreased absolutely from 0.34 kPa to 0.26 kPa and P 0.1/Pi max from 0.06 to 0.04. Due to the small sample size (n = 2) the difference did not reach significance (Table S3).

The subgroup analysis of the pooled subjects with cardiocirculatory conditions (arterial hypertension n = 23, chronic heart failure n = 1, coronary artery disease n = 1) showed an absolute increase of FEV_1_ (+11.6%, p<0.001), VC (+7.5, p<0.001), FEV_1_/VC (+3.0, p = 0.046) and an absolute increase of P 0.1 (+0.07 kPa, p = 0 006) (Table S3).

## Discussion

There is no doubt that obesity is associated with a large variety of diseases including cardiopulmonary dysfunction [46], [47], and is a major contributor to morbidity [2], [3] and mortality [3], [4], [46]. However, surprisingly little is known about the influence of obesity on cardiac and pulmonary function in obese individuals who have none or very mild symptoms. Importantly, it is unclear whether dietary weight loss programs lead to improved cardiopulmonary function similar to what has been reported for surgical weight loss programs [34].

The study cohort consisted of severely obese individuals the majority presenting asymptomatic in NYHA Class I without dyspnea. Although some subjects had comorbidities such as arterial hypertension, diabetes and dyslipidemia, which may have contributed to the increase in LV wall thickness, they had no obvious end organ damage. In our cohort we detected functional pulmonary and cardiac abnormalities at baseline which significantly improved after weight loss.

Obesity can lead to OHS and contribute to elevated pulmonary artery pressure in OHS. However, not all obese subjects develop OHS [12] and not all patients with OHS show PH [13]. Although in our cohort maximal inspiratory mouth pressures was normal [38], [39] and there was only a mild decrease of airflow the subjects showed an impairment of P 0.1 and P0.1/Pi max [38], [39]. The increased P0.1 and P0.1/Pi max in asymptomatic subjects highlights the increased respiratory load early on in obese subjects. The results are in line with data reporting abnormal breathing patterns in obese subjects with no history of pulmonary disease [15]. Despite the mild changes of ventilatory parameters at baseline the correlation of improving VC and FEV1 with loss of body fat further strengthens the assumption that obesity has a major impact on ventilatory function in these individuals.

Although an association of asthma and overweight has been proposed [17], [18], there is still an ongoing debate on the degree of the interaction between obesity and asthma [21], [30]. Our subjects showed a significant correlation of weight and body fat with pulmonary functional impairment. The importance of body fat distribution for ventilatory function has been described previously [17]. Our subjects showed a correlation of waist-to-hip ratio with vital capacity and TLC indicating a predominant role of central obesity for ventilatory abnormalities.

Obesity has a high impact on cardiac morbidity and mortality [1], [4]. An association between obesity and pulmonary mortality has also been proposed [46]. Cardiovascular disease [47], obstructive sleep apnea [9] and hypoventilation syndrome [10] are associated with obesity. Recent data suggest a contribution of obesity to the development of hypoventilation associated severe pulmonary hypertension [11], an independent influence of body weight on pulmonary hypertension in diastolic left ventricular function [8] and also obesity cardiomyopathy [6], [7].

The evaluation of myocardial function in obese subjects needs to differentiate hypercontractility [6], [7] in early stages of obesity, impaired left ventricular function in patients affected by manifest cardiomyopathy and additional parameters of myocardial function such as left and right myocardial performance index [48], [49], annular tissue velocity and annular valve flow parameters reflecting diastolic function. Despite left ventricular ejection fraction and TAPSE being normal, we found elevated left and right myocardial performance index as an early sign of cardiac (diastolic and systolic) functional distress [43], [48], [49]. Myocardial wall thickness of the interventricular septum and posterior wall was mildly enlarged [42]. Echocardiography in severely obese individuals is difficult to perform, but all echocardiographic procedures were performed by a highly experienced physician. Due to absence of detectable tricuspid valve insufficiency, right ventricular systolic pressure could not be estimated in the majority of tests. However, right ventricular outflow acceleration time (RVOT AT) could be measured in nearly all subjects. Therefore RVOT AT is a helpful non-invasive parameter for a qualitative analysis of pulmonary blood flow and its reduction is associated with decreasing pulmonary artery pressure and vascular resistance [50]. The acceleration time of the right ventricular outflow tract was mildly decreased in our cohort, suggesting the presence of increased pulmonary artery pressure and pulmonary vascular resistance [50].

The positive correlation of myocardial wall thickness and the negative correlation of pulmonary outflow tract acceleration time with the waist- to-hip ratio suggest an influence of central obesity on cardiac function and pulmonary perfusion. The morphological cardiac and functional cardiopulmonary changes may have been mild, but the improvement of these parameters as well as the correlation of weight and body fat loss with myocardial wall thickness and improvement of myocardial performance index highlight the clinical significance of the results. The correlation between decreased body fat and improved vital capacity and FEV_1_ following weight loss further suggests that the abnormalities are part of an early pathophysiological process leading ultimately to disease in obese individuals.

Weight loss is strongly recommended for patients with obesity related diseases such as obstructive sleep apnea [51], [52], but the effect of early intervention on lung and cardiac function in asymptomatic obese subjects is less clear. It has previously been shown that surgical induced weight loss has positive effects on cardiac function [31], [53], [54], [55], [56]. Syed reported a decrease of myocardial mass, but no significant improvement of functional parameters following a structured program combining diet, exercise and surgical procedures. However, in this study left myocardial performance index and acceleration time in the right ventricular outflow tract was not investigated [34]. There are only few reports on dietary induced weight loss and its effect on cardiac and pulmonary function. Reduction of body fat improved lung function after a Mediterranean diet [32]. A modest improvement of lung volumes at rest after a moderately successful dietary weight loss was shown and a relationship with the amount of chest fat was discussed [33].

In our study, the participants achieved a remarkable weight loss, that was substantially higher than in previous reports [32], [33] leading to a highly significant increase in lung volumes, ventilatory flow parameters and a decrease of respiratory load. We found significant improvement of early indicators of disturbed myocardial performance such as LIMP and MAPSE. Additionally our subjects had an improvement of myocardial wall thickness following successful weight loss. We noticed an improvement of the RVOT AT following weight loss in our study subjects. This may indicate reduced pulmonary vascular resistance and improved blood flow following a dietary induced weight loss.

Our cohort consisted of asymptomatic or almost asymptomatic obese patients. There were only few patients with relevant comorbidities. Subjects with a history of asthma and sleep apnea showed an improvement of pulmonary function comparable to the improvement of the whole cohort. The lack of significance of FEV_1_/FVC in the asthmatics is the result of an increase of FEV_1_ and vital capacity and of the small sample size of subjects with comorbidities. Although there is a clear signal towards an improvement of lung function in the few subjects with a history of asthma and OSA and cardiocirculatory conditions, this should be investigated in further studies especially addressing subjects with comorbidities.

Our study has several limitations. Not all subjects seen at baseline were examined after the weight loss program, but only patients with data available at both baseline and three month follow-up period were included into the analysis of changes in each parameter. Bioelectrical impedance analysis was not performed in all patients. However, the robustness of our data still suggest that asymptomatic individuals without end organ damage entering a structured weight loss program show mild cardiac and pulmonary functional impairment associated with weight and body fat. This can be reversed solely by a structured dietary induced weight loss.

### Conclusion

In a cohort of severely obese asymptomatic adults we found evidence for early functional pulmonary and cardiac distress. Lung volumes and left ventricular myocardial performance index were negatively correlated to body weight. Airway flow is negatively and respiratory load is positively correlated to body fat. Myocardial wall thickness is positively correlated to body weight. Right ventricular outflow tract acceleration time and myocardial wall thickness is correlated to waist-to-hip ratio. A structured dietary program resulted in significant improvement of ventilatory and myocardial parameters in these obese individuals strongly suggesting that aggressive weight loss programs as early as possible may be able to prevent cardiopulmonary morbidity.

## Supporting Information

Table S1 **Results of Bioelectric Impedance analysis at baseline.**
(DOCX)

Table S2 **History of comorbidities.**
(DOCX)

Table S3 **Weight, BMI and pulmonary function test data of subjects with a history of asthma, obstructive sleep apnea or cardiocirculatory conditions.** (arterial hypertension n = 23, chronic heart failure n = 1, coronary artery disease n = 1).
(DOCX)
