# Supplementary material for: Pulmonary and Cardiac Function in Asymptomatic Obese Subjects and Changes following a Structured Weight Reduction Program: A Prospective Observational Study
Source: PLoS One. 2014 Sep 18;9(9):e107480. doi: 10.1371/journal.pone.0107480 (PMC4169401; doi:10.1371/journal.pone.0107480)
Supplement: Table S1 — Results of Bioelectric Impedance analysis at baseline. (DOCX) [file pone.0107480.s001.docx]

| **Parameter** |  | **N= 74, Value Mean ± SD** |
| --- | --- | --- |
| **Bioelectrical impedance analysis** | | |
| Metabolic base rate (Kcal) | 58 | 1756,4 ± 293,9 |
| Phase angle (°) | 58 | 6,02 ± 0,72 |
| Body fluid (l) | 58 | 50,7 ± 11,5 |
| Lean body mass (kg) | 58 | 69,3 ± 15,7 |
| ECM (kg) | 58 | 33,2 ± 7,2 |
| BCM (kg) | 58 | 36,1 ± 9,3 |
| ECM/BCM-Index | 58 | 0,94 ± 0,13 |
| Cell amount (%) | 58 | 51,9 ± 3,4 |
| Body fat (kg) | 58 | 55,8 ± 16,6 |
| Body fat (%) | 58 | 44,4 ± 8,4 |
| Body fat corrected (kg) | 58 | 57,3 ± 16,8 |
| Body fat corrected (males) | 58 | 32.7 ± 15.1 |
| Body fat corrected (females) | 58 | 41.7 ± 13.2 |

Table S1: Results of Bioelectric Impedance analysis at baseline.
