# Supplementary material for: Pulmonary and Cardiac Function in Asymptomatic Obese Subjects and Changes following a Structured Weight Reduction Program: A Prospective Observational Study
Source: PLoS One. 2014 Sep 18;9(9):e107480. doi: 10.1371/journal.pone.0107480 (PMC4169401; doi:10.1371/journal.pone.0107480)
Supplement: Table S2 — History of comorbidities. (DOCX) [file pone.0107480.s002.docx]

Table S2: History of comorbidities.

| Arterial hypertension | 32 |
| --- | --- |
| Diabetes | 13 |
| hyperlipidemia | 10 |
| History of hypothyroidism, controlled | 10 |
| Asthma | 9 |
| Neurological disorder | 7 |
| Depression | 5 |
| Obstructive sleep apnea | 3 |
| Coronary artery disease | 2 |
| Hyperthyroidism | 1 |
| Kidney disease | 1 |
| Rhythm disorder | 1 |
| History of pulmonary embolism | 1 |
