# Supplementary material for: Pulmonary and Cardiac Function in Asymptomatic Obese Subjects and Changes following a Structured Weight Reduction Program: A Prospective Observational Study
Source: PLoS One. 2014 Sep 18;9(9):e107480. doi: 10.1371/journal.pone.0107480 (PMC4169401; doi:10.1371/journal.pone.0107480)
Supplement: Table S3 — Weight, BMI and pulmonary function test data of subjects with a history of asthma, obstructive sleep apnea or cardiocirculatory conditions. (arterial hypertension n = 23, chronic heart failure n = 1, coronary artery disease n = 1). (DOCX) [file pone.0107480.s003.docx]

| Subjects with a history of asthma | | | | | |
| --- | --- | --- | --- | --- | --- |
| Parameter | n | Baseline (Mean±SD) | Follow-Up (Mean±SD) | Absolute Change (Mean±SD) | p-value |
| Weight (kg) | 7 | 136.1±32.6 | 103.0±24.8 | -33.1±6.4 | 0.002 |
| BMI (kg/m^2^) | 7 | 44.6±10.2 | 33.9±8.6 | -10.7±4.6 | 0.001 |
| FEV_1_ (% pred) | 5 | 93.8±31.1 | 107.6±31.2 | +13.8±2.3 | <0.001 |
| VC (% pred) | 5 | 101.6±15.2 | 112.2±15.5 | +10.6±4.2 | < 0.01 |
| FEV_1_/VC (%) | 5 | 70.7±17.9 | 71.8±14.4 | +1.1±3.8 | 0.55 |
| RV (% pred) | 5 | 120.2±38.8 | 104.4±45.9 | -15.8±33.5 | 0.35 |
| P 0.1 (kPa) | 6 | 0.38±0.18 | 0.26±0.13 | -0.125±0.12 | 0.47 |
| TLCO-SB (% pred) | 4 | 90.8±11.5 | 100.0±13.3 | +9.2±3.8 | 0.02 |
| TLC_He_ (% pred) | 4 | 92.8±17.2 | 102.8±18.1 | +10.0±5.0 | 0.03 |
|  | | | | | |
| Subjects with a history of sleep apnea | | | | | |
| Parameter | n | Baseline (Mean±SD) | Follow-Up (Mean±SD) | Absolute Change (Mean±SD) | p-value |
| Weight (kg) | 3 | 160.0±33.4 | 122.0±30.4 | -38.0±7.2 | 0.01 |
| BMI (kg/m^2^) | 3 | 48.8±14.8 | 37.3±12.8 | -11.5±2.9 | 0.02 |
| FEV_1_ (% pred) | 3 | 82.3±35.9 | 94.7±33.7 | +12.3±3.1 | 0.02 |
| VC (% pred) | 3 | 90.0±10.4 | 100.3±9.0 | +10.3±1.5 | <0.01 |
| FEV_1_/VC (%) | 3 | 66.1±22.4 | 77.8±27.6 | +11.7±13.6 | 0.28 |
| P 0.1 (kPa) | 3 | 0.34±0.29 | 0.26±0.18 | -0.08±0.15 | 0.45 |
|  | | | | | |
| Subjects with a cardio circulatory conditions | | | | | |
| Parameter | N | Baseline (Mean±SD) | Follow-Up (Mean±SD) | Absolute Change (Mean±SD) | p-Value |
| Weight (kg) | 24 | 126.7±22.0 | 98.9±16.8 | -27.8±11.0 | <0.001 |
| BMI (kg/m^2^) | 24 | 42.1±6.8 | 32.9±5.8 | -9.2±3.3 | <0.001 |
| FEV_1_ (% pred) | 24 | 97.2±20.0 | 109.5±18.9 | 12.3±9.1 | <0.001 |
| VC (% pred) | 24 | 96.4±13.6 | 103.7±14.8 | +7.3±7.4 | <0.001 |
| FEV_1_/VC (%) | 24 | 77.3±9.9 | 80.1±9.6 | +2.8±6.6 | 0.049 |
| MEF 25 (% perd) | 24 | 71.3±34.6 | 84±36.5 | +12.7±23.1 | 0.013 |
| ITGV (% pred) | 24 | 94.4±15.9 | 114.1±20.1 | +19.8±17.3 | <0.001 |
| SR tot (% pred) | 24 | 120.8±76.1 | 91.7±39.3 | -29.1±50.9 | 0.01 |
| TLC (He) (% pred) | 17 | 88.0±10.0 | 94.0±9.1 | +5.9±7.1 | 0.003 |
| P 0.1(kPa) | 19 | 0.32±0.14 | 0.25±0.13 | -0.07±0.10 | 0.006 |

Table S3: Weight, BMI and pulmonary function test data of subjects with a history of asthma, obstructive sleep apnea or cardiocirculatory conditions (arterial hypertension n= 23, chronic heart failure n=1, coronary artery disease n=1).
